# Feasibility of a Mobile Cognitive Intervention in Childhood Absence Epilepsy

**DOI:** 10.3389/fnhum.2016.00575

**Published:** 2016-11-15

**Authors:** Peter Glynn, Soyong Eom, Frank Zelko, Sookyong Koh

**Affiliations:** ^1^Feinberg School of Medicine, Northwestern University, ChicagoIL, USA; ^2^Epilepsy Center, Department of Pediatrics, Ann & Robert H. Lurie Children’s Hospital of Chicago, Feinberg School of Medicine, Northwestern University, ChicagoIL, USA; ^3^Epilepsy Research Institute, College of Medicine, Yonsei UniversitySeoul, South Korea; ^4^Department of Psychiatry and Behavioral Science, Feinberg School of Medicine, Northwestern University, ChicagoIL, USA; ^5^Department of Child and Adolescent Psychiatry, Ann & Robert H Lurie Children’s Hospital of Chicago, ChicagoIL, USA; ^6^Department of Pediatrics, Emory Children’s Center, Children’s Healthcare of Atlanta, School of Medicine, Emory University, AtlantaGA, USA

**Keywords:** cognitive rehabilitation, childhood absence epilepsy, attention, iPad, Constant Therapy

## Abstract

Children with childhood absence epilepsy (CAE) frequently present with cognitive comorbidities and school performance concerns. The present study evaluated the feasibility of an intervention for such comorbidities using a mobile cognitive therapy application on an iPad. Eight children with CAE and school concerns aged 7–11 participated in a 4-week intervention. They were asked to use the application for 80 min per week (20 min/day, 4 times/week). Parents and children completed satisfaction surveys regarding the application. Participants were evaluated before and after the intervention using the Cognitive Domain of the NIH Toolbox and by parental completion of the Behavioral Rating Inventory of Executive Function. All eight patients completed the study, using the iPad for an average of 78 min/week. Children and parents reported high satisfaction with the application. Though a demonstration of efficacy was not the focus of the study, performance improvements were noted on a processing speed task and on a measure of fluid intelligence. An iPad based cognitive therapy was found to be a feasible intervention for children with CAE.

## Introduction

Approximately 345,000 children in the United States are diagnosed with epilepsy (National Survey of Children with Special Health Care Needs NS-CSHCN, 2009/10) with childhood absence epilepsy (CAE) accounting for approximately 10–17% of all cases (Masur et al., 2013). While CAE is generally considered to be relatively benign – occurring in children with normal development, intelligence and neurological examination – many children with CAE have dificulties with problem solving, language, and attention (Conant et al., 2010; D’Agati et al., 2012). Up to 43% of children have been documented as having a linguistic deficit, particularly discourse and semantic deficits (Caplan et al., 2008). Despite this significant percentage, problems with attention have been particularly well-documented and are the dominant deficit in CAE (Caplan et al., 2008; Conant et al., 2010; Vega et al., 2010; D’Agati et al., 2012; Cerminara et al., 2013). Drug therapy may play a role in these difficulties (Glauser et al., 2010, 2013). Regardless of whether it is due to medications, epilepsy itself, or both, children with CAE struggle in school in a way that their peers do not.

Childhood absence epilepsy is typically treated with ethosuximide, valproate, lamotrogine, or levetiracetam, with ethosuximide generally considered the first line agent (Matricardi et al., 2014). Yet even when seizure control is achieved, deficits in attention and executive function persist (Masur et al., 2013; Matricardi et al., 2014). Cognitive rehabilitation is one strategy that can be used to target these deficits. Cognitive rehabilitation focuses on restoring impaired function via repeated skills practice and facilitating compensation for deficits through instruction in cognitive strategies (Kesler et al., 2011). Cognitive rehabilitation has been well-studied as an intervention following traumatic brain injury or stroke, with sufficient evidence to support its use as a therapeutic too (Cicerone et al., 2000; Cappa et al., 2005). However, the study of cognitive rehabilitation in epilepsy has been limited, especially in children, and clinically only 23% of children with CAE receive any kind of intervention to help with their school struggles (Caplan et al., 2008).

Given their well-described cognitive comorbidities, their low rate of utilization of therapy services, and their overall school struggles, children with CAE are natural candidates for cognitive rehabilitation therapy. That it can now be delivered remotely via technology is particularly appealing because of how interested and fluent in technology children are. A rehabilitation program delivered on a mobile tablet platform, such as the iPad (Apple, Inc., Cuppertino, CA, USA), seems particularly attractive for that reason.

Several cognitive training tools, including CogMed, Lumosity, ACTIVATE and Constant Therapy (CT), are now available on the iPad (Mayas et al., 2014). CT was developed specifically as a rehabilitation tool and it has shown promising results in adults with post-stroke aphasia (Des Roches et al., 2014). The application delivers a customizable battery of cognitive and language rehabilitation therapies via the iPad and provides real-time feedback to the user. The software logs and displays data regarding performance (accuracy, latency) and use (tasks completed, time spent). The library of tasks spans the domains of reading, writing, naming, attention, memory, and problem solving (see Cognitive Training under Section “Materials and Methods” below for more detail). Using CT as a representative model, the present study investigated whether a mobile cognitive therapy protocol is a feasible intervention for children with CAE and school concerns. We sought to determine if children would use the application frequently with minimal prompting and if they would enjoy using it. A secondary objective of the study was to explore the potential neurocognitive benefit of using the application for 4 weeks.

## Materials and Methods

### Participants

Children with CAE and school concerns were recruited from the Epilepsy Center at Ann and Robert H. Lurie Children’s Hospital of Chicago. The parents of all potential participants had explicitly expressed concern over cognition or school performance; this was necessary for inclusion in the study. It was not required that potential participants had undergone formal assessment of cognitive function or received a clinical diagnosis of cognitive deficit. Among the 20 potential candidates who were contacted by phone regarding study participation, eight (four boys) ultimately enrolled. Of those who declined to participate, most simply never called back after initial contact and never responded to additional phone calls. A few expressed concerns about the time commitment required for the study, particularly during the summer months. Two additional participants verbally agreed to start the study but did not come to their scheduled sessions.

The ages of the participants ranged from 7 to 11 years old (9.8 ± 1.7). The age of seizure onset ranged from 2 to 9 years (4.8 ± 2.1). At the time of enrollment, seven of the eight participants were seizure free, with a mean seizure-free duration of 2.8 years (*SD* = 2.3). Four children were being treated with valproic acid, two with lamotrigine, and two had been on ethosuximde but currently were not receiving an AED (**Table 1**). Despite many of the patients being in remission of CAE and some heterogenieity in AED history, all participants shared a diagnosis of CAE and current parent concerns over cognition or school performance, which were the primary criteria for inclusion in this feasibility study. All children and parents were either English monolinguals or English/Spanish bilinguals. Informed consent was obtained from the parents of each participant in accordance with policies set forth by the Ann and Robert H. Lurie Children’s Hospital Institutional Review Board.

**Table 1:** Demographic data of participants with Childhood Absence Epilepsy.

Subject	Gender	Age	Sz onset	Sz free	AED	iPad
1	M	11	9	1	VPA	Yes
2	M	10	6	4	None	No
3	F	7	4	No	VPA	No
4	M	7	3	4	None	No
5	F	10	2	2	VPA	Yes
6	F	11	4	2	LTG	Yes
7	F	11	4	6	LTG	Yes
8	M	11	6	2	VPA	No

*Sz onset, age of onset of seizures in years; Sz free, number of years without clinically evident absence seizures; AED, anti-epileptic drugs; VPA, valproic acid; LTG, lamotrigine; iPad, patient’s own iPad used for study.*

Five of the participants had access to an iPad at home, but one of the iPads did not support the CT application. Thus four children borrowed an iPad for the duration of the study. For one participant who did not have wifi network data access at home, a 3G data plan was supplied for the duration of the study. Compensation, beyond reimbursment for parking expenses, was not provided. The iPads used in this study were obtained through an iPad scholarship program mediated by Boston University. Use of CT was provided free of charge by Constant Therapy Inc. All eight children completed the study.

### Study Design

The study had three components: a pre-intervention visit to the Clinical Research Unit of Lurie Children’s Hospital (visit 1), a 4-week home intervention with the therapy application, and a post-intervention visit to the Clinical Research Unit (visit 2).

#### Visit 1

After obtaining consent from each participant’s parent, participants completed the Cognitive Domain of the NIH Toolbox (Gershon et al., 2010) and parents completed the Behavioral Rating Inventory of Executive Function (BRIEF) (Gioia et al., 2000). If the participant required an iPad to use for the study, one was provided with the application installed. If a participant did not require an iPad, the application was downloaded and installed on the participant’s iPad. Training in how to log into the application, access assigned tasks, and access other tasks within the application was provided to both children and parents.

#### Cognitive Training

CT was selected as the cognitive training tool because of the first author’s previous experience with the application. Each participant was asked to use the cognitive training program for 20 min per day, four times per week, for a total of 4 weeks. If they desired, the children were free to use the application more than that. The CT software logged the amount of time spent using the application. Once per week during the training period, an investigator called home to gage the experience of each child. The investigator inquired as to whether tasks were at the right difficulty level, whether the child was too frustrated or too bored, and whether there were specific tasks that the child enjoyed and wanted to work on more or specific tasks that the child did not enjoy. These conversations were generally brief- no more than 5 min long.

For consistency, each participant began the same six training tasks at the same level of difficulty, assigned 10 items of each task type. As inattention is the dominant deficit in CAE, the six initial tasks were selected to target attention or working memory, which requires attention to selectively encode and manipulate information (Fougnie, 2008). The six initial tasks, detailed in **Table 2**, were Symbol Matching, Card Slapjack, Flanker, Picture Matching, Picture N-back, and Pattern Recreation. For visual depiction of these tasks, please see **Supplementary Figures S1**–**S6**.

**Table 2:** The six initial tasks selected for attention or working memory training.

Task name	Level	Contents
Symbol Matching	1	A task of visual attention requiring the subject to identify all instances of a symbol prototype in a grid filled with distractor symbols.
Card Slapjack	1	A task of visual attention requiring the subject to tap the iPad screen whenever the prototype card (i.e., 3 of clubs) is presented in a stream of distractor cards (i.e., 3 of hearts).
Flanker	1	A task of visual attention requiring the subject to indicate what direction the central, target arrow, is pointing among a field of distractor arrows.
Picture Matching	1	A task of visual working memory requiring the subject to match identical pictures presented in a grid.
Picture N-back	1	A visual working memory task requiring the subject to tap the iPad screen when the picture presented is identical to the previous picture presented (n-1).
Pattern Recreation	1	A visual working memory task that lights up a series of four squares of a grid in a given order and asks the participant to recreate the order by tapping the correct boxes on the screen.

Based on each individual’s performance, the level of difficulty and the nature of the tasks were modified. For instance, some of these tasks (Symbol Matching, Picture Matching, Picture N-back, and Pattern Recreation) had multiple levels of difficulty. When a participant demonstrated mastery at a given level, the next level of difficulty was presented. Mastery was determined by performance accuracy; in most instances a single score of 100% or two consecutive scores over 90% prompted an increase in difficulty level. In some cases clinical judgment was used to increasing the difficulty level after a single score above 90% or a series of three or more scores just below 90%. On some tasks, such as the Flanker task and Card Slapjack task, only one level of difficulty is available. When mastery was achieved on those tasks, they continued to be included in future training sessions to assess for improvements in reaction time.

When the highest level of a task was completed, or when reaction time plateaued, these initial tasks were replaced with new tasks. The new tasks fell into two categories: (1) tasks selected by the investigators to continue to target attention, language, or problem solving, and (2) tasks that the individual participants expressed a preference for in weekly phone calls. Some children experimented with other tasks in the application outside of their assigned tasks and discovered other tasks they enjoyed in this way. Additional tasks were often chosen with variety of material in mind, in an effort to prevent boredom and burnout. As such, those tasks were as varied as arithmetic, math word problems, reading passage and other language tasks, and several different tasks focusing on attention and working memory. In total, participants collectively completed 50 different kinds of tasks, spanning domains of attention, memory, processing speed, language, and executive function.

#### Visit 2

Participants came in for the post-intervention visit within a week of completion of the 4th week of therapy. During the second visit, participants again completed the Cognitive Domain of the NIH Toolbox. The first author administered pre and post-treatment NIH Toolbox assessments, as well as the cognitive training, and as such was not blinded to diagnosis or study goals. Parents also completed the BRIEF post-intervention, and both participants and parents were asked to fill out debriefing questionnaires that addressed their satisfaction with Constant Therapy and with the idea of a mobile cognitive intervention generally.

#### Measures

The Cognitive Domain of the NIH Toolbox is a series of six computer tasks that evaluates attention, memory, language, and executive skills. These tasks include a picture vocabulary test, the flanker inhibitory control and attention test, the dimensional card sort test, a list sorting working memory test, a pattern comparison processing speed test, and an oral reading recognition test. This assessment takes about 45 min to administer, and provides scores on the individual tasks and a number of composite measures. The BRIEF is a pencil and paper questionnaire which provides parental ratings of participants’ everyday attention and executive skills, and takes about 15 min for parents to complete. Satisfaction with the CT application was assessed by two separate debriefing surveys, one filled out by the parent and one filled out by the child. The participant survey specifically addressed enjoyment, ease of use, and favorite and least favorite aspects of the application for each individual child. The parent survey was more wide ranging and addressed overall satisfaction, satisfaction with content and ease of use, perceptions of child’s use and enjoyment, parental likes and dislikes, and willingness to use a similar application in the future.

### Statistical Analysis

Descriptive statistics (means, standard error, and frequency counts) were used to characterize subjects’ utilization of CT over the study period and ratings of satisfaction with the application as expressed by subjects and their parents. Wilcoxon signed rank tests were used to compare pre- vs. post-intervention performances on NIH Cognitive Toolbox parameters and parent ratings on the BRIEF in preliminary explorations of the potential efficacy of the CT intervention.

## Results

### Feasibility

**FIGURE 1:**
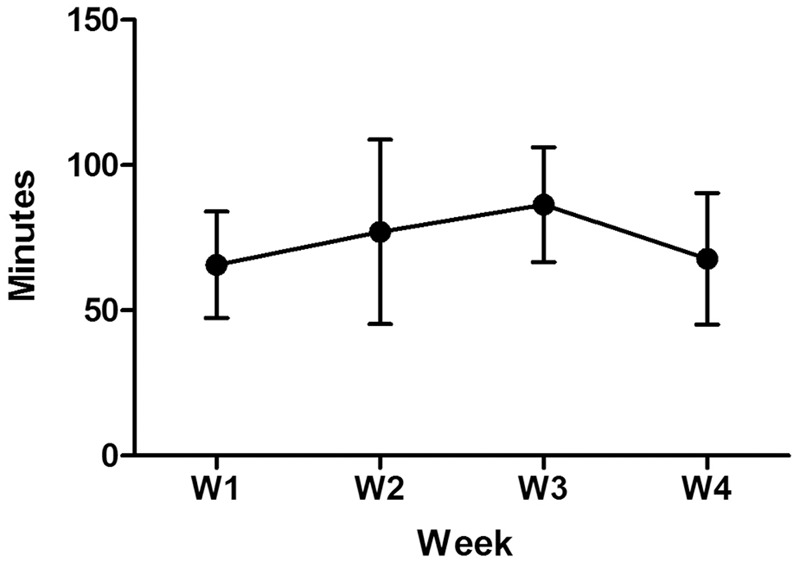
**Weekly usage of CT over 4 weeks.** The average group data shows the mean use varied from 65 to 85 min per week. Variation of usage from week to week as well as inter-subject variability were noted as reflected by standard error bars.

Of the eight children who were enrolled, all completed the study, including visit 1, the cognitive training, and visit 2. The average duration of use was 78 min per week (**Table 3**). During this time, participants completed varying numbers of therapy tasks, with totals ranging from 234 to 1687, and an average weekly total of 207 tasks. Two children used the application for significantly more minutes per week (110 and 221), while two other children used the application for significantly fewer minutes per week (14 and 28). The other four children clustered around 60 min per week. When averaged across the group, use was steady across the duration of the trial period, between 65 and 85 min per week (**Figure 1**). However, there was a great deal of variability in individual use from week to week, and between different subjects.

**Table 3:** Compliance of participants with cognitive intervention.

Subject	Time spent^a^		Number of tasks^b^	
	Total	Per week	Total	Per week
1	245.61	61.4	919	229.8
2	56.84	14.2	234	58.5
3	110.00	27.5	298	74.5
4	438.80	109.7	861	215.3
5	227.55	56.9	822	205.5
6	884.08	221.0	1687	421.8
7	246.62	61.7	669	167.3
8	272.07	68.0	1145	286.3
Average		77.5		207.4

*^a^Time spent on tasks in minutes. 80 min/week was prescribed amount; ^b^Number of tasks completed.*

The children expressed high levels of satisfaction with the application. All eight reported that they enjoyed using it, and six reported that they would use it for fun without prompting. All eight reported that it was easy to use and that they believed it helped with their cognitive abilities (**Table 4**). When asked what they liked most and least about the application, the children identified their favorite and least favorite tasks. These responses varied and did not reveal a clear pattern.

**Table 4:** Satisfaction with the cognitive therapy.

Subjects	Yes	No
(1) Did you like using the app?	8	0
(2) Would you use it for fun if no one asked you to?	6	2
(3) Do you think it helped you improve?	8	0
(4) Was it easy to use?	8	0

**Parents**		**Rating^∗^**

(1) How satisfied were you with the therapy application as a tool for your child?		4.4
(2) How satisfied were you with the quality of its content?		4.5
(3) How well did the content address your child’s needs?		4.1
(4) How satisfied were you with the app’s usability?		5
(5) How “kid-friendly” was it?		4.6

*^∗^Average of parental ratings on a scale from 0 to 5, where 0 is “not at all satisfied” and 5 is “very satisfied.”*

The parents of participants expressed similar levels of satisfaction with the application (**Table 4**). Parents were asked to rate the application on a zero to five Likert scale (0 = not at all, 5 = very much) on a series of questions. They indicated that they were satisfied with the application’s usability (mean rating = 5), with how “kid-friendly” it was (4.6), with the quality of its content (4.5), with it as a therapy tool for their child (4.4), and with how well the content addressed their child’s needs (4.1). When asked their perspective of their child’s experience with the cognitive therapy, all eight parents reported that their child enjoyed using the application. Six said that their child was able to use the application independently – one reported that their child needed help starting the application and accessing the tasks, while another reported needing help with a specific math task. Two parents reported that their child got bored with the application. When asked how much parental supervision their child needed, six parents reported that they needed none, two parents reported that they needed some; no parents reported their child needing “a lot” of supervision. Two parents reported noticing functional changes in their child, one noting improvements in memory, another in reading comprehension.

When given the chance to describe the application’s strengths, a few themes emerged. Four parents described the application/technology as engaging for their child. Three described the application as easy to use, and two described it as enjoyable/exciting to use. When describing its weaknesses, three parents reported that the level of difficulty was not always right for their child; sometimes it was too hard, and others too easy. Three parents also reported that their child had difficulty getting motivated to start the therapy program. However, when asked if they would be interested in using a similar tablet intervention in the future, all eight parents said yes.

### Efficacy

Two external measures were used to explore the potential efficacy of the 4 week intervention with Constant Therapy – parent ratings on the BRIEF and participants’ peformance on the Cognitive Domain of the NIH Toolbox.

The BRIEF revealed no statistically significant differences in pre–post comparisons of parental ratings of executive function. It is notable, however, that the mean reported Global Executive Composite both pre (63.1) and post (62.1) intervention were more than a standard deviation above the population mean of 50 (SD: 10), indicating executive difficulties in the study cohort.

On the Cognitive Domain of the NIH Toolbox, two statistically significant differences were found when comparing pre-intervention to post-intervention scores (**Table 5**, *p* < 0.05 non-parametric Wilcoxon signed rank tests). Measures showing significant improvement were the pattern comparison processing speed test and the Cognition Fluid Composite. The Cognition Fluid Composite, which is based upon performance indices from all the fluid ability measures (flanker task, dimensional change card sort, picture sequence memory task, list sorting, and pattern comparison), is a measure of fluid intelligence, or the ability to use logic to solve new problems, independent of previously acquired knowledge. Of note, the post–pre comparisons for the composites and the dimensional change card sort task were based upon only seven post-intervention values because data from one task were lost in one subject.

**Table 5:** Impact of mobile cognitive intervention on performance upon the cognition domain of the NIH Toolbox.

	Mean (SD)		*P*
	Pre-intervention (*n* = 8)	Post-intervention^a^ (*n* = 8)	
Cognition Total Composite^b^	88.8 (11.2)	96.2 (14.3)	0.063
Cognition Crystallized Composite	99.8 (13.8)	102.0 (12.6)	0.401
Cognition Fluid Composite^b^	88.5 (13.5)	96.7 (15.3)	0.018^∗^
Cognition Early Childhood Composite^b^	92.1 (11.6)	99.2 (13.7)	0.028^∗^
Picture Vocabulary	92.3 (11.5)	97.4 (15.6)	0.208
Flanker Inhibitory Control and Attention	88.1 (16.4)	91.5 (17.6)	0.161
List Sorting Working Memory	93.0 (21.8)	93.3 (10.3)	0.600
Dimensional Change Card Sort^b^	88.1 (9.9)	90.4 (5.8)	0.310
Pattern Comparison Process Speed	81.2 (12.1)	89.9 (15.3)	0.018^∗^
Picture Sequence Memory	90.3 (9.4)	97.3 (11.9)	0.050
Oral Reading Recognition	91.5 (19.6)	94.1 (10.3)	0.484

*^a^Non-parametric, Wicoxon signed rank test in SPSS between baseline and follow-up evaluation at 5 weeks; ^∗^*p* < 0.05. ^b^Note that post-therapy values for the Cognition Total, Cognition Fluid, and Cognition Early Childhood Composites, as well as for the Dimensional Change Card Sort only include seven subjects due to a Toolbox malfunction.*

## Discussion

This study demonstrated the feasibility of a mobile cognitive intervention in children with CAE and school concerns. The prescribed amount of therapy time was 80 min per week. The observed average use was 78 min per week, with a large variance. Subject 2 was an outlier in this regard, using the application significanlty more than the rest of the study group. As a group, use across the duration of the study period was relatively steady. Given that children and parents were largely left to freely determine how much to use the application, and that there were no incentives to use the application beyond that level and no consequences for using it less, the level of participants’ engagement with the cognitive therapy application over a 4-week period seemed sufficient for the purpose of clinical intervention. The variability in weekly use among and between individuals will be an important point in future research.

Both parents and children alike reported high levels of satisfaction with the cognitive training. Children found it enjoyable and 75% (6/8) reported that they would use the application for fun, even if no one asked them to. Both parents and children found it easy to use. Parents reported high levels of satisfaction with the quality of the content of the application while a few questioned whether the content addressed their child’s needs. A goal for a future study will be to address how the application’s specific content could be modified to address each child’s individual needs. Most important, all eight parents reported that they would be interested in using a similar mobile cognitive therapy in the future.

Taken together with the usage data, the satisfaction questionnaire responses support the feasibility of mobile cognitive intervention as a therapy for children with CAE.

This is significant because of the dearth of the data on the use of cognitive rehabilitation in epilepsy, particularly childhood epilepsy. A recent review by Farina et al. (2015) reported only nine studies of cognitive rehabilitation in epilepsy between 1974 and 2012. The studies are not homogenous with regard to sample population or length/type of intervention. Several were limited by small sample sizes or lacked randomization or control groups. Most important, none of the studies were carried out with children. Given their well-described deficits across the cognitive and language domains, lack of utilization of therapy services, and the feasibility demonstrated by the present study, children with CAE appear to be a population that would benefit from mobile cognitive rehabilitation therapies provided efficacy can be established.

While feasibility was the primary focus of this study, some preliminary data regarding efficacy were obtained. Comparing pre and post-intervention BRIEF ratings revealed no differences in parental reporting of executive function. This is not entirely surprising, as the intervention duration was only 4 weeks, and BRIEF is typically used as a broad spectrum measure of executive skills sampled over a longer periof of time (Kurowski et al., 2014). Other measurement tools such as Conners parent Assessment Report may have been more senstive to capture subtle changes resulting from short-term intervention (Steiner et al., 2014). However, those tools address ADHD symptomatology, which was not defined as a specific focus of this investigation. The data from the Cognitive Domain of the NIH Toolbox do suggest a few differences, most notably increases in processing speed and on a measure of fluid intelligence. The increase in fluid intelligence is potentially significant, as one of the main deficits associated CAE is in problem solving. Similarly, processing speed is a concern in childhood epilepsy and may be associated with antiepileptic drug therapy (Lagae, 2006). It should be noted, however, that the freedom afforded to children to explore tasks outside of the prescribed training tasks does confound the suggestion of improvement in fluid intelligence, as these additional tasks may have contributed to that improvement.

While encouraging, these results need to be interpreted cautiously. First, the sample size is small (*n* = 8, and only seven in the case of post-intervention fluid composite scores). Second, the satisfaction questionnare ratings of CT and BRIEF could have been biased favorably by the expectation of parents and children for the computer games to be therapeutic tools. However, we would expect less of an effect upon measures from the NIH Toolbox. Third, like many other cognitive measures, the NIH Toolbox Cognitive battery is subject to practice effects, particularly over a relatively short, 4-week period (Weintraub et al., 2014). It is possible that the significant efficacy results are confounded by a short term test-retest effect. Inclusion of a control group across 4 weeks without the intervention (CT) would be essential to prove efficacy. Further, the study would be more impactful if the subjects had a proven learning deficit and if there were pre and post school tests to show whether the effectiveness of cognitive therapy can be translated to real school performance. In future efficacy studies of iPad based cognitive therapy, random assignment to a therapy group and a control group should be a priority.

In summary, we have shown that the use of iPad based mobile cognitive rehabilliation is feasible with potential benefit in children with CAE and school concerns. The efficacy of cognitive therapy in children with epilepsy warrants more rigorous study with a larger sample size and a treatment control group. Mobile cognitive intervention has potential to be utilized as a component of a comprehensive treatment program for children with CAE.

## Ethical Approval

This study was reviewed and approved by the Institutional Review Board of Ann and Robert H. Lurie Children’s Hospital of Chicago (#2014-15731).

## Author Contributions

PG and SE conducted pre and post-intervention visits. PG remotely administered cognitive therapy via Constant Therapy. Data analysis was carried out by SE. Preparation of the manuscript was completed by, in order of contribution, PG, SE, and equally FZ and SK. Study design and conceptualization was carried out by PG, FZ, and SK equally, and SE.

## Conflict of Interest Statement

PG contributed to the development of the Constant Therapy application and has the potential to receive compensation in the event that Boston University profits from the sale of its equity in CT in the future. All the other authors declare that the research was conducted in the absence of any commercial or financial relationships that could be construed as a potential conflict of interest.

## Supplementary Material

FIGURE S1 **Pattern Recreation.** Boxes illuminate sequentially in a given pattern, then the subject is asked to recreate that pattern from memory. This depicts the end result after a subject has successfully recreated a five box sequence.

FIGURE S2 **N-back Task.** A picture is displayed on the screen. The subject is asked to remember that picture before a new picture is displayed. When *n* = 1, the subject taps the screen if the picture displayed is the same as the previous item. When *n* = 2, he/she taps if the picture currently displayed is the same as the item prior to the last item, and so on.

FIGURE S3 **Picture Matching.** A grid of boxes is displayed. The subject taps a box to visualize the picture it contains. The subject then searches for a matching picture in the grid. If he/she identifies the same picture with consecutive taps, the items remain displayed. If not, the images disappear and the subject searches again.

FIGURE S4 **Flanker Task.** An image is displayed that contains a string of arrow heads. The subject is asked to indicate the direction that the red arrowhead is pointing. Distractor arrowheads may point the same or different directions than the red arrow.

FIGURE S5 **Slap Jack Task.** The subject is presented with a prototype image. A series of images, some matching the prototype and some distractors, is displayed. The subject is asked to tap the screen whenever the prototype image is displayed.

FIGURE S6 **Symbol Matching.** The subject is shown a prototype symbol and a grid of symbols, some that match the prototype and some distractors. The subject is asked to indicate every symbol that matches the prototype.

## References

[B1] Caplan R. Siddarth P. Stahl L. Lanphier E. Vona P. Gurbani S. (2008). Childhood absence epilepsy: behavioral, cognitive, and linguistic comorbidities. *Epilepsia* 49 1838–1846. 10.1111/j.1528-1167.2008.01680.x 18557780

[B2] Cappa S. F. Benke T. Clarke S. Rossi B. Stemmer B. van Heugten C. M. (2005). EFNS guidelines on cognitive rehabilitation: report of an EFNS task force. *Eur. J. Neurol.* 12 665–680. 10.1111/j.1468-1331.2005.01330.x 16128867

[B3] Cerminara C. D’Agati E. Casarelli L. Kaunzinger I. Lange K. W. Pitzianti M. (2013). Attention impairment in childhood absence epilepsy: an impulsivity problem? *Epilepsy Behav.* 27 337–341. 10.1016/j.yebeh.2013.02.022 23537619

[B4] Cicerone K. D. Dahlberg C. Kalmar K. Langenbahn D. M. Malec J. F. Bergquist T. F. (2000). Evidence-based cognitive rehabilitation: recommendations for clinical practice. *Arch. Phys. Med. Rehabil.* 81 1596–1615. 10.1053/apmr.2000.19240 11128897

[B5] Conant L. L. Wilfong A. Inglese C. Schwarte A. (2010). Dysfunction of executive and related processes in childhood absence epilepsy. *Epilepsy Behav.* 18 414–423. 10.1016/j.yebeh.2010.05.010 20656561

[B6] D’Agati E. Cerminara C. Casarelli L. Pitzianti M. Curatolo P. (2012). Attention and executive functions profile in childhood absence epilepsy. *Brain Dev.* 34 812–817. 10.1016/j.braindev.2012.03.001 22459253

[B7] Des Roches C. A. Balachandran I. Ascenso E. M. Tripodis Y. Kiran S. (2014). Effectiveness of an impairment-based individualized rehabilitation program using an iPad-based software platform. *Front. Hum. Neurosci.* 8:1015 10.3389/fnhum.2014.01015 PMC4283612 25601831

[B8] Farina E. Raglio A. Giovagnoli A. R. (2015). Cognitive rehabilitation in epilepsy: an evidence-based review. *Epilepsy Res.* 109 210–218. 10.1016/j.eplepsyres.2014.10.017 25524861

[B9] Fougnie D. (2008). “The relationship between attention and working memory,” in *New Research on Short-Term Memory* ed. Johansen B. N. (Hauppauge, NY: Nova Science Publishers, Inc.) 1–45.

[B10] Gershon R. C. Cella D. Fox N. A. Havlik R. J. Hendrie H. C. Wagster M. V. (2010). Assessment of neurological and behavioural function: the NIH Toolbox. *Lancet Neurol.* 9 138–139. 10.1016/S1474-4422(09)70335-r7 20129161

[B11] Gioia G. A. Isquith P. K. Guy S. C. Kenworthy L. (2000). Behavior rating inventory of executive function. *Child Neuropsychol.* 6 235–238. 10.1076/chin.6.3.235.3152 11419452

[B12] Glauser T. A. Cnaan A. Shinnar S. Hirtz D. G. Dlugos D. Masur D. (2010). Ethosuximide, valproic acid, and lamotrigine in childhood absence epilepsy. *N. Engl. J. Med.* 362 790–799. 10.1056/NEJMoa0902014 20200383 PMC2924476

[B13] Glauser T. A. Cnaan A. Shinnar S. Hirtz D. G. Dlugos D. Masur D. (2013). Ethosuximide, valproic acid, and lamotrigine in childhood absence epilepsy: initial monotherapy outcomes at 12 months. *Epilepsia* 54 141–155. 10.1111/epi.12028 23167925 PMC3538883

[B14] Kesler S. R. Lacayo N. J. Jo B. (2011). A pilot study of an online cognitive rehabilitation program for executive function skills in children with cancer-related brain injury. *Brain Inj.* 25 101–112. 10.3109/02699052.2010.536194 21142826 PMC3050575

[B15] Kurowski B. G. Wade S. L. Kirkwood M. W. Brown T. M. Stancin T. Taylor H. G. (2014). Long-term benefits of an early online problem-solving intervention for executive dysfunction after traumatic brain injury in children: a randomized clinical trial. *JAMA Pediatr.* 168 523–531. 10.1001/jamapediatrics.2013.5070 24781374 PMC4113596

[B16] Lagae L. (2006). Cognitive side effects of anti-epileptic drugs. The relevance in childhood epilepsy. *Seizure* 15 235–241. 10.1016/j.seizure.2006.02.013 16563808

[B17] Masur D. Shinnar S. Cnaan A. Shinnar R. C. Clark P. Wang J. (2013). Pretreatment cognitive deficits and treatment effects on attention in childhood absence epilepsy. *Neurology* 81 1572–1580. 10.1212/WNL.0b013e3182a9f3ca 24089388 PMC3806916

[B18] Matricardi S. Verrotti A. Chiarelli F. Cerminara C. Curatolo P. (2014). Current advances in childhood absence epilepsy. *Pediatr. Neurol.* 50 205–212. 10.1016/j.pediatrneurol.2013.10.009 24530152

[B19] Mayas J. Parmentier F. B. Andres P. Ballesteros S. (2014). Plasticity of attentional functions in older adults after non-action video game training: a randomized controlled trial. *PLoS ONE* 9:e92269 10.1371/journal.pone.0092269 PMC3960226 24647551

[B20] National Survey of Children with Special Health Care Needs NS-CSHCN (2009/10). *Data query from the Child and Adolescent Health Measurement Initiative, Data Resource Center for Child and Adolescent Health website*. Available at: www.childhealthdata.org [accessed November 1 2015].

[B21] Steiner N. J. Frenette E. C. Rene K. M. Brennan R. T. Perrin E. C. (2014). In-school neurofeedback training for ADHD: sustained improvements from a randomized control trial. *Pediatrics* 133 483–492. 10.1542/peds.2013-2059 24534402

[B22] Vega C. Vestal M. DeSalvo M. Berman R. Chung M. Blumenfeld H. (2010). Differentiation of attention-related problems in childhood absence epilepsy. *Epilepsy Behav.* 19 82–85. 10.1016/j.yebeh.2010.06.010 20674507 PMC2943027

[B23] Weintraub S. Dikmen S. S. Heaton R. K. Tulsky D. S. Zelazo P. D. Slotkin J. (2014). The cognition battery of the NIH toolbox for assessment of neurological and behavioral function: validation in an adult sample. *J. Int. Neuropsychol. Soc.* 20 567–578. 10.1017/S1355617714000320 24959840 PMC4103959

