# Supplementary figures and images for: Feasibility of a Mobile Cognitive Intervention in Childhood Absence Epilepsy
Source: Front Hum Neurosci. 2016 Nov 15;10:575. doi: 10.3389/fnhum.2016.00575 (PMC5108758; doi:10.3389/fnhum.2016.00575)

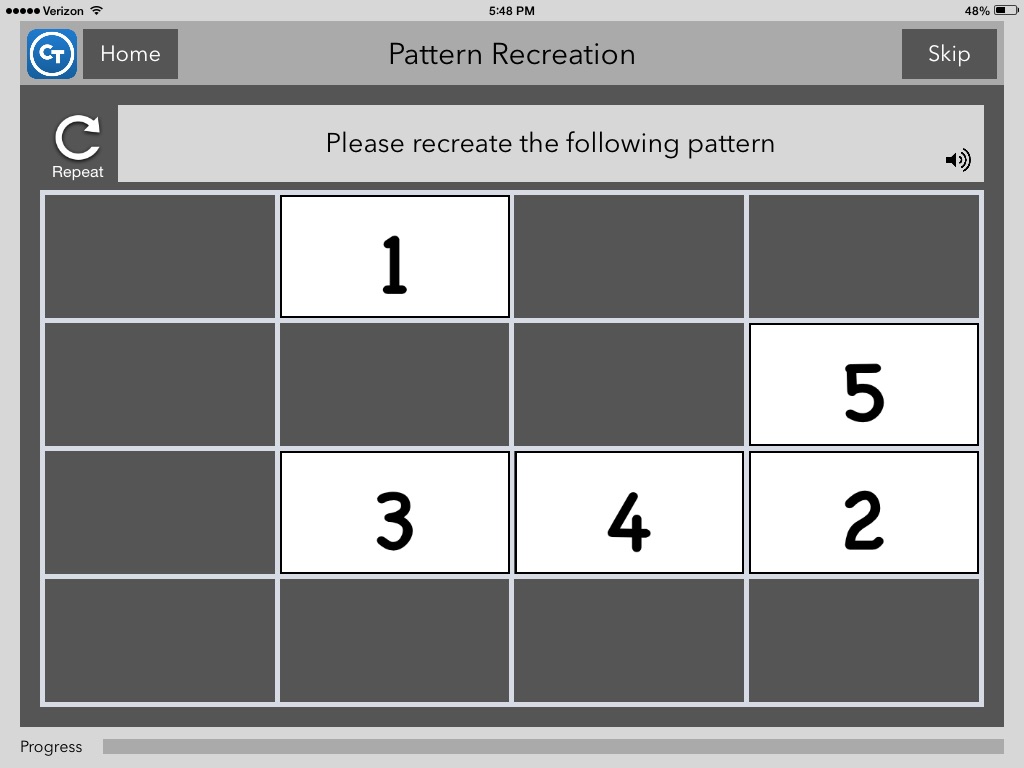

Supplement: FIGURE S1 — Pattern Recreation. Boxes illuminate sequentially in a given pattern, then the subject is asked to recreate that pattern from memory. This depicts the end result after a subject has successfully recreated a five box sequence. [file Image_1.JPEG]

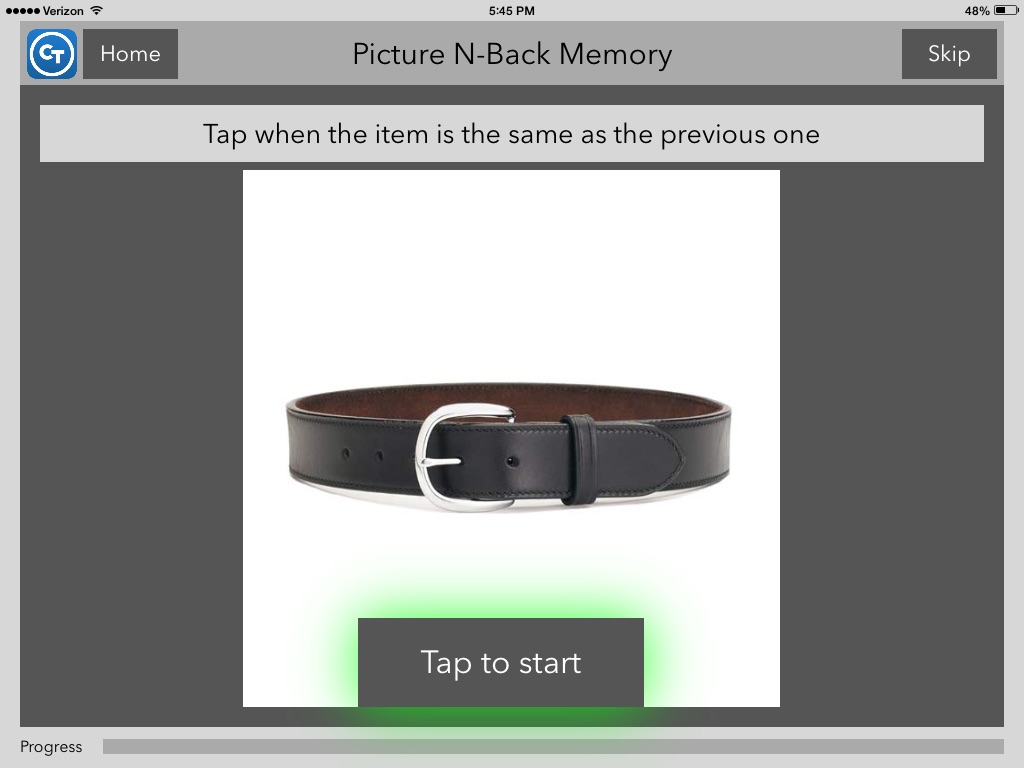

Supplement: FIGURE S2 — N-back Task. A picture is displayed on the screen. The subject is asked to remember that picture before a new picture is displayed. When n = 1, the subject taps the screen if the picture displayed is the same as the previous item. When n = 2, he/she taps if the picture currently displayed is the same as the item prior to the last item, and so on. [file Image_2.JPEG]

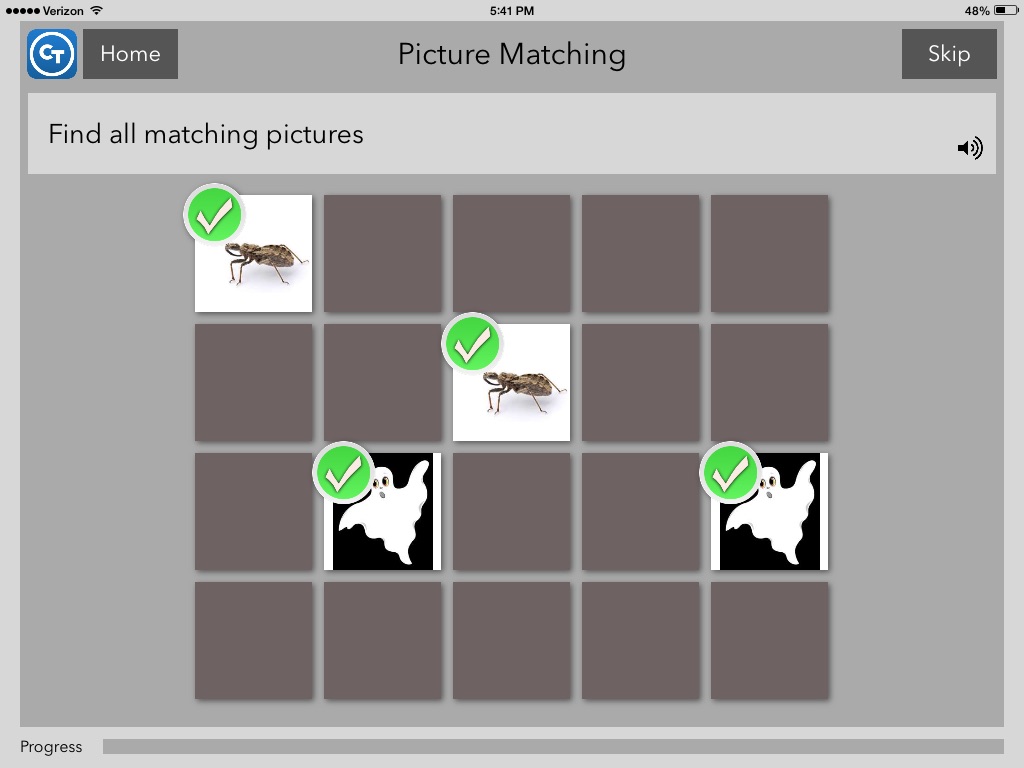

Supplement: FIGURE S3 — Picture Matching. A grid of boxes is displayed. The subject taps a box to visualize the picture it contains. The subject then searches for a matching picture in the grid. If he/she identifies the same picture with consecutive taps, the items remain displayed. If not, the images disappear and the subject searches again. [file Image_3.JPEG]

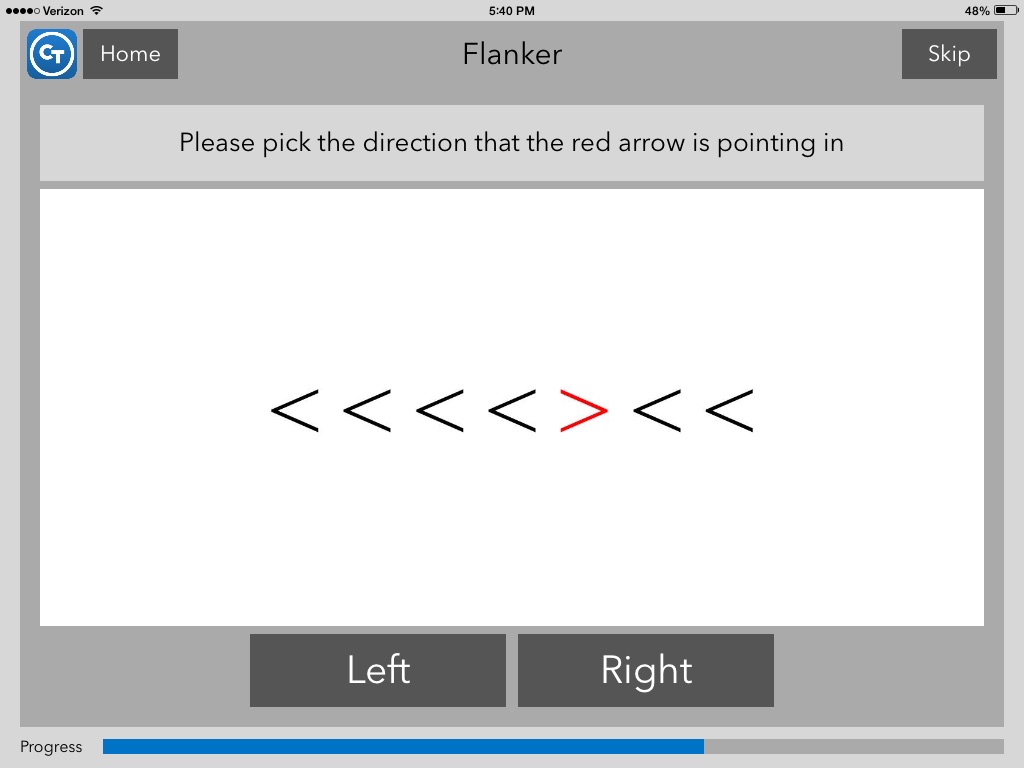

Supplement: FIGURE S4 — Flanker Task. An image is displayed that contains a string of arrow heads. The subject is asked to indicate the direction that the red arrowhead is pointing. Distractor arrowheads may point the same or different directions than the red arrow. [file Image_4.JPEG]

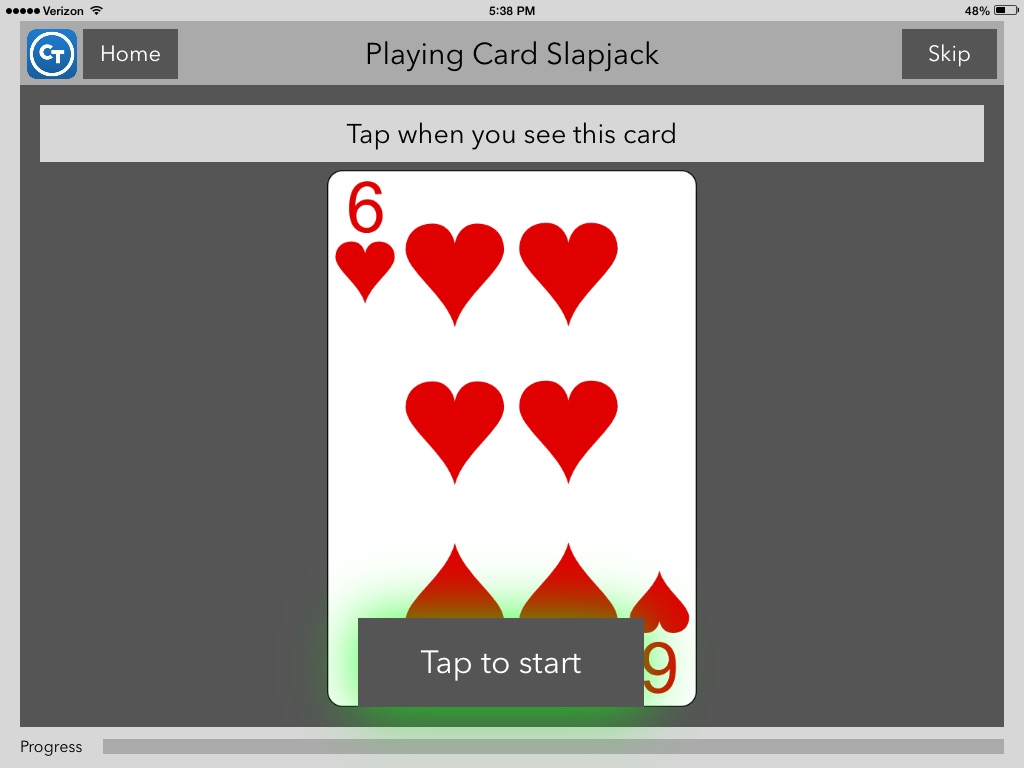

Supplement: FIGURE S5 — Slap Jack Task. The subject is presented with a prototype image. A series of images, some matching the prototype and some distractors, is displayed. The subject is asked to tap the screen whenever the prototype image is displayed. [file Image_5.JPEG]

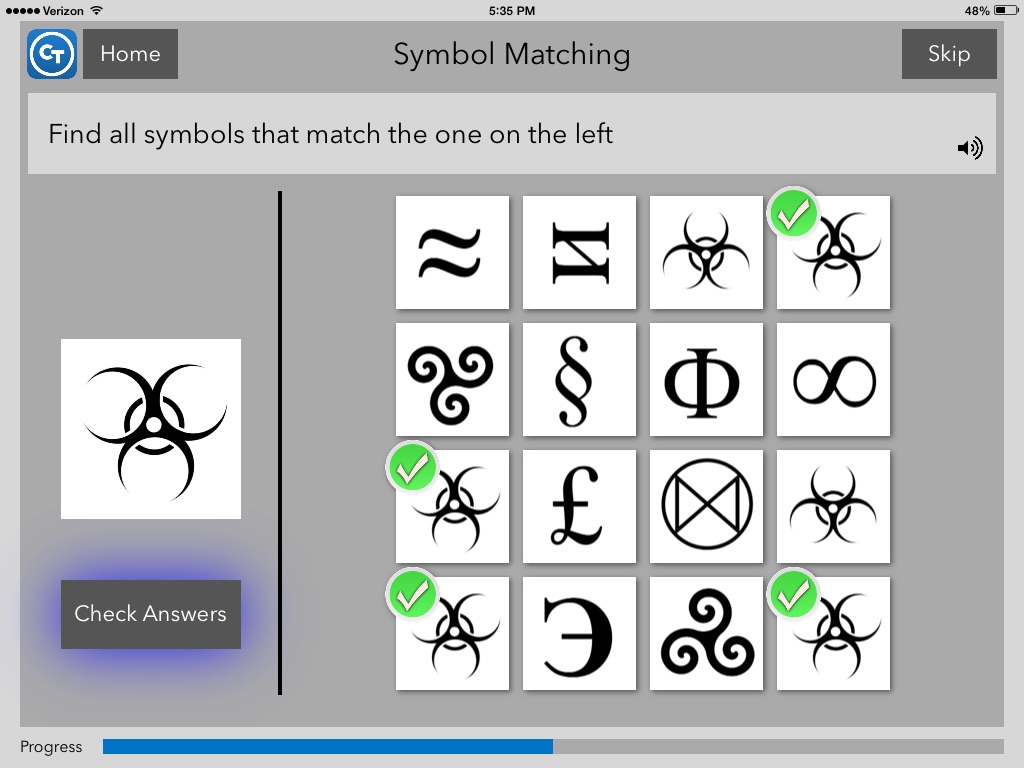

Supplement: FIGURE S6 — Symbol Matching. The subject is shown a prototype symbol and a grid of symbols, some that match the prototype and some distractors. The subject is asked to indicate every symbol that matches the prototype. [file Image_6.JPEG]
